# Novel Cysteine-Sparing Hypomorphic *NOTCH3* A1604T Mutation Observed in a Family With Migraine and White Matter Lesions

**DOI:** 10.1212/NXG.0000000000000584

**Published:** 2021-04-22

**Authors:** Snjolaug Arnardottir, Francesca Del Gaudio, Stefanos Klironomos, Eike-Benjamin Braune, Ariane Araujo Lombraña, Daniel V. Oliveira, Shaobo Jin, Helena Karlström, Urban Lendahl, Christina Sjöstrand

**Affiliations:** From the Division of Neurology (S.A., S.K., C.S.), Department of Clinical Neuroscience, Karolinska Institutet; Department of Cell and Molecular Biology (F.D.G., E.-B.B., A.A.L., S.J., U.L.), Karolinska Institutet; and Department of Neurobiology, Care Sciences and Society (D.O., H.K., U.L.), Karolinska Institutet, Stockholm, Sweden.

## Abstract

**Objective:**

To conduct a clinical study of a family with neurologic symptoms and findings carrying a novel *NOTCH3* mutation and to analyze the molecular consequences of the mutation.

**Methods:**

We analyzed a family with complex neurologic symptoms by MRI and neurologic examinations. Exome sequencing of the *NOTCH3* locus was conducted, and whole-genome sequencing was performed to identify *COL4A1*, *COL4A2*, and *HTRA1* mutations. Cell lines expressing the normal or NOTCH3^A1604T^ receptors were analyzed to assess proteolytic processing, cell morphology, receptor routing, and receptor signaling.

**Results:**

Cerebral autosomal dominant arteriopathy with subcortical infarcts and leukoencephalopathy (CADASIL) is the most common hereditary form of cerebral small vessel disease (SVD) and caused by mutations in the *NOTCH3* gene. Most CADASIL mutations alter the number of cysteine residues in the extracellular domain of the NOTCH3 receptor, but in this article, we describe a family in which some members carry a novel cysteine-sparing NOTCH3 mutation (c.4810 G>A, p.Ala1604Thr). Two of 3 siblings heterozygous for the NOTCH3^A1604T^ mutation presented with migraine and white matter lesions (WMLs), the latter of a type related to but distinct from what is normally observed in CADASIL. Two other members instead carried a novel COL4A1 missense mutation (c.4795 G>A; p.(Ala1599Thr)). The NOTCH3^A1604T^ receptor was aberrantly processed, showed reduced presence at the cell surface, and less efficiently activated Notch downstream target genes.

**Conclusions:**

We identify a family with migraine and WML in which some members carry a cysteine-sparing hypomorphic *NOTCH3* mutation. Although a causal relationship is not established, we believe that the observations contribute to the discussion on dysregulated Notch signaling in cerebral SVDs.

Cerebral dominant arteriopathy with subcortical infarcts (cerebral autosomal dominant arteriopathy with subcortical infarcts and leukoencephalopathy [CADASIL]; OMIN No 125310) is the most common monogenic form of cerebral small vessel disease (SVD). Patients with CADASIL develop early-onset vascular dementia, relatively symmetrical white matter lesions (WMLs) with microbleeds, and lacunar ischemic infarcts as well as dilated perivascular spaces.^1-6^

CADASIL is caused by mutations in the *NOTCH3* gene,^7-9^ and most CADASIL mutations result in an altered number of cysteine residues in the NOTCH3 extracellular domain (ECD). A disease-specific feature of CADASIL pathology is deposition of so-called granular osmiophilic material (GOM) around vascular smooth muscle cells (VSMC), and GOM contains the mutated NOTCH3 ECD.^9-12^

Although classical CADASIL *NOTCH3* mutations are cysteine altering, less is known about other, cysteine-sparing, mutations and their potential role in neurologic disease.^13^ In Notch signaling, ligands on one cell activate transmembrane Notch receptors on a juxtaposed cell.^14^ Ligand-receptor interaction results in proteolytic processing of the Notch receptor, eventually liberating the Notch intracellular domain (Notch ICD). Notch ICD increases activation of Notch downstream genes such as *Hes* and *Hey* genes.^14,15^ Notch signaling is critical for VSMC function and homeostasis, with a loss of VSMC in Notch3^−/−^ mouse brains.^16-19^ Although some CADASIL mutations appear to be signaling-neutral,^20^ the NOTCH3^C455R^ CADASIL mutation resulted in reduced signaling.^21^

To gain insights into cysteine-sparing *NOTCH3* mutations, in this report, we identify a family with a novel missense *NOTCH3* mutation and reveal that the mutation affects Notch receptor processing and signaling.

## Methods

### Clinical Investigations

The index patient was referred to the neurology department because of severe migraine and WML shown on MRI of the brain. A genetic screen for CADASIL was performed within clinical routine; peripheral blood from the index patient and additional family members was drawn for sequencing of the exons in the *NOTCH3* gene (Centogene Lab, Rostock, Germany). Whole-genome sequencing (WGS) of the index patient and 2 other family members (mother and older sister) was conducted (see below), and potential mutations in the collagen type IV alpha1 (*COL4A1*), collagen type IV alpha2 (*COL4A2*), and Htra serine protease 1 (*HTRA1*) genes were analyzed. MRI of the brain was performed in several family members, and images were interpreted by an experienced neuroradiologist and further examined within the research group. Physical and neurologic examination was performed on the index patient and on some other family members. A questionnaire on headache and migraine, history of vascular events, vascular risk factors, and comorbidities was filled in by all participating family members.

### Standard Protocol Approvals, Registrations, and Patient Consents

All participants gave their written informed consent. The study was approved by the local ethics committee.

### Data Availability

Because of their sensitive nature, a subset of the data generated in this study is available from the corresponding author on request, given that the request is not in conflict with the General Data Protection Regulation directive 2016/679.

### WGS and Bioinformatic Analysis

Standard 30× WGS and bioinformatic analysis were performed at Clinical Genomics, SciLifeLab, Stockholm, using the Illumina NovaSeq 6000 platform. Single nucleotide variants were called using Mutation Identification Pipeline (github.com/henrikstranneheim/MIP). Variants were filtered, removing variants with a frequency over 1% in the general population. A constructed gene panel (*COL4A1, COL4A2, HTRA1*, and *NOTCH3*) was analyzed regarding variants affecting coding regions and/or splicing. Three individuals in the family were analyzed.

### Notch3^A1604T^ Cloning

From a plasmid expressing a full-length human *NOTCH3* wild-type receptor gene (tagged with c-Myc and histidine tags in the C-terminus), a fragment encompassing the 1604 residue was removed by AfeI and Bsu36I digestion. The fragment was mutated from an alanine to threonine at the 1604 residue (from a 4810-G to a 4810-A nucleotide) by site-directed mutagenesis and then inserted back into the original full-length NOTCH3 plasmid. PCR primers used for the cloning are listed in table e-2, links.lww.com/NXG/A415, in the online-only data supplement.

### Cell Culture and Treatments

HEK293T cells were purchased from the American Type Culture Collection. HEK293T cells and the resulting NOTCH3-expressing cell lines were maintained as described.^22^ To block Notch signaling, cells were treated with 10 µM of the γ-secretase inhibitor DAPT (Merck Millipore, Darmstadt, Germany) for 24 hours. To activate Notch signaling, cells were mechanically dissociated with accutase (Life Technologies, Thermo Fisher Scientific, Inc., Waltham, MA) and seeded on 24-well plates precoated overnight with Jagged1-Fc or Delta-like4-Fc or only with Fc fragment, as the control (R&D System, Minneapolis, MN). Proteasome activity was blocked by 20 μM MG132 (Sigma-Aldrich, Saint Louis, MO) for 4 hours before harvesting the cells.

### Cell Growth Curve

HEK 293T(ΔN1-N3)^WT^ and HEK 293T(ΔN1-N3)^A1604T^ cells were seeded in triplicate in 24-well plates at the same starting density and harvested after 24, 48, or 72 hours. For each time point, the cells were washed in phosphate-buffered saline (PBS) (Gibco, Thermo Fisher Scientific, Inc., Waltham, MA), dissociated with accutase (Life Technologies), and counted with Trypan blue (Gibco) in Dual Chamber Counting Slides (Bio-Rad, Hercules, CA) using the TC20 counter (Bio-Rad).

### Quantitative Real-Time PCR

Quantitative real-time PCR (qRT-PCR) was performed as previously described^22^ and on a Bio-Rad CFX96 System (Bio-Rad). Primers used in qRT-PCR are listed in table e-4, links.lww.com/NXG/A415, in the online-only data supplement.

### Cell Surface Biotinylation

Cell surface biotinylation was performed as previously described^23^ with the following modification: cell lysates were homogenized by passing each lysate 5 times through a 19-G syringe and subsequently centrifuged for 10 minutes at 4°C at 10,000*g* to remove cellular debris.

### Immunocytochemistry

Twenty-four thousand cells per well were seeded in 8-well chamber slides (BD Falcon, BD Bioscience, San José, CA) and incubated for 24 hours. Cells were then fixed with glyoxal solution (Sigma-Aldrich), as previously described.^24^ Subsequently, the cells were permeabilized with 0.3% Triton-X (Sigma-Aldrich) in PBS for 10 minutes at room temperature (RT) and washed in PBS-0.1% TritonX. Cells were blocked in 3% bovine serum albumin (BSA) at RT for 30 minutes and incubated with primary antibodies diluted in 1% BSA-0.1% Triton-X in PBS for 1 hour at RT. Cells were washed 3 times and incubated with fluorescence-conjugated secondary antibodies in 1% BSA-0.1% TritonX for 30 minutes at RT. Cells were washed 3 times and probed with DAPI (Roche, Basel, Switzerland) to stain for DNA. After 1 wash in only PBS, slides were mounted with Vectashield Antifade Mounting Medium (Vector Labs, Burlingame, CA). Confocal images were taken with a Leica SP8 microscope (Leica Microsystem, Heerbrugg Switzerland). All antibodies used are listed in table e-3, links.lww.com/NXG/A415, in the online-only data supplement.

For cell transfections, CRISPR/Cas9 genome editing, Western blot, and statistical analysis, see Supplementary Information (links.lww.com/NXG/A415).

### Modeling of the Protein Stability Changes Resulting From the A1604T Mutation

To predict the change in the protein stability on mutation, we used the (DUET) server.^25^ The images of the wild-type and mutated proteins are obtained by the online tool Structure (National Center for Biotechnology Information [NCBI]). The PDB file representing the 3D structures of the mutated NOTCH3 NRR domain is available (e-7 in the online-only data supplement, links.lww.com/NXG/A415).

## Results

### Clinical Presentation of a Family Carrying a Novel NOTCH3^A1604T^ Mutation

**Figure 1:**
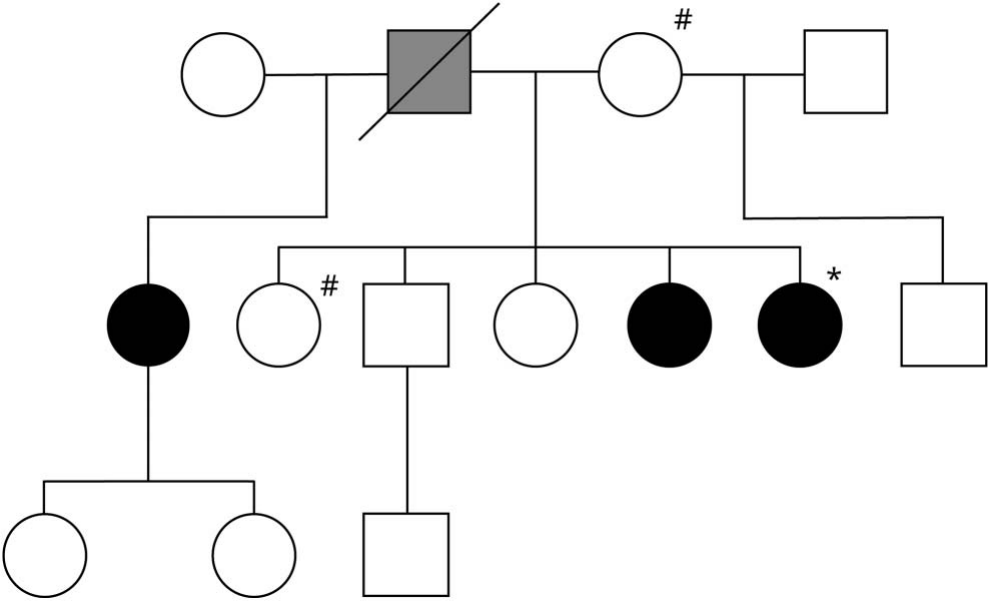
Family Pedigree of the Family Carrying the *NOTCH3^A1604T^* Mutation Open circle = normal woman; black circle = woman heterozygous for *NOTCH3^A1604T^*; open square = normal man; black square = man heterozygous for *NOTCH3^A1604T^*. Gray square = man who likely was heterozygous for *NOTCH3^A1604T^*. *Index patient. #Family members carrying the *COL41A^A1599T^* mutation.

In this study, we report the discovery of a family carrying a novel type of *NOTCH3* mutation: a missense mutation, c.4810 G>A, resulting in an alanine to threonine transition at amino acid residue 1604 (A1604T). The family pedigree and the segregation of the A1604T mutation are presented in figure 1. The female index patient had migraine with aura with frequent attacks and severe pain since age 18 years. MRI analysis of the brain, first conducted at age 42 years, showed WML, most prominent in the frontal area. Multiple sclerosis was initially suspected, but follow-up investigation including CSF analysis showed no signs of inflammation. Furthermore, MR angiography of neck and cerebral arteries performed at age 43 years showed no abnormalities. Based on the clinical symptoms of migraine with aura and WML on MRI, the index patient was again referred to the neurology department and analyzed for *NOTCH3* mutations. The index patient was found to be heterozygous for a novel A1604T mutation in the *NOTCH3* gene, and the analysis was extended to other members of the family. Subsequent to a thorough investigation for depression, the index patient was diagnosed with a neuropsychiatric disorder. Mild polyneuropathy was also found following neurophysiologic investigation. Histopathologic investigation of a muscle biopsy did not indicate any pathology (data not shown), ruling out mitochondrial disease. The MRI analysis revealed features that were similar to but not typical of most patients with CADASIL (see below). MRI was performed several times over the years, and lesions progressed mildly over time, but with no obvious progression after inclusion in this study at age 52 years. CT angiography of cerebral arteries performed at age 52 years showed no abnormalities.

The index patient had 4 siblings and 2 half-siblings, 1 from each parent (figure 1). The younger sister of the index patient, at age 51 years on examination, was also heterozygous for the A1604T mutation. Clinical examination and a neurologic examination showed that she had migraine with aura with frequent attacks and severe pain for many years. A CT scan of the brain performed at age 42 years revealed hypodensities of the white matter, most prominent in the frontal area. MRI of the brain after study inclusion showed the same type and distribution of WMLs, but less prominent than in the index patient (see below).

**Figure 2:**
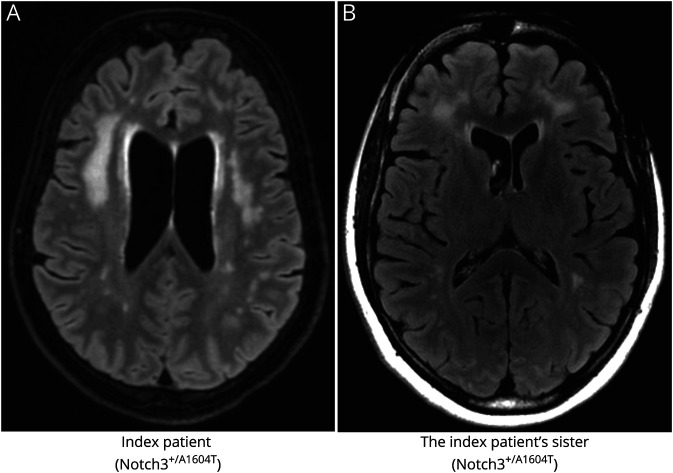
MRIs From 2 Siblings Heterozygous for the NOTCH3^A1604T^ Mutation The index patient (A) was subjected to axial 3D fluid-attenuated inversion recovery (FLAIR). Confluent WMLs were found predominantly in the frontal subcortical and deep white matter. No lesions where found in the anterior temporal region and only a few small lesions in capsula externa. The index patient's heterozygous sister (B) was subjected to axial 2D FLAIR. A similar lesion localization as in the index patient was observed but with somewhat smaller lesion load. Bifrontal predilection, with no lesions in the anterior temporal area, and few lesions in capsula externa were also observed. WML = white matter lesion.

The MRI findings in the index patient and her younger sister, both heterozygous for the A1604T mutation, were consistent with features typically found in CADASIL although some dissimilarities were observed (figure 2; an MRI from a patient with CADASIL is shown in figure e-1, links.lww.com/NXG/A415). As is typical for CADASIL,^5,6^ there was a predominance of WML in the frontal and periventricular areas with sparing of the subcortical U-fibers. The frontal lesions were confluent in both the index patient and the *NOTCH3^A1604T^*-carrying sister. The parietal deep white matter was the second most affected area. However, contrary to classical CADASIL, there was only mild or no involvement of the temporal pole and external capsule. No lesions were observed in the basal ganglia. The pons was mildly affected in the index patient and the heterozygous sister, and no microbleeds were found. There were no periventricular microvascular changes typical for CADASIL in the index patient. There were neither signs of ischemic or hemorrhagic stroke nor evidence of vasculitis in any of the analyzed family members.

The father of the index patient, who must have had the A1604T mutation because it was observed in 3 of the children but not in his wife (see below), deceased at age 66 years and had experienced an intracerebral bleeding at age 47 years (figure 1). The mother of the index patient, age 77 years, was negative for the A1604T mutation. She had migraine with aura at younger age; in addition, she had hypertension and mild polyneuropathy. MRI of the brain showed some WML, but of a microangiopathic type, which was distinct from WML seen in the index patient and the younger sister (figure e-2, links.lww.com/NXG/A415). The oldest sister of the index patient, age 58 years, was negative for the A1604T mutation. She had migraine with aura earlier in life and hypertension since about age 30 years and obstructive sleep apnea syndrome. MRI of the brain showed WML, but it was neither typical for CADASIL nor similar to those in the index patient. The MRI of the brain of the oldest sister showed confluent WMLs almost equally distributed in the frontal and parietal regions and, to a lesser extent, in the posterior temporal, periventricular, pontine, and deep cerebellar white matter, with sparing of the subcortical U-fibers. There were also some T2-hyperintense bilateral foci in the basal ganglia and capsula externa. There were no infarcts or evidence of larger hemorrhagic lesions. No susceptibility-sensitive sequences were available; therefore, the presence or absence of microbleeds could not be ascertained (figure e-2). The half-sister (same father) of the index patient, age 58 years, was heterozygous for the A1604T mutation. She had headache, most likely migraine without aura, earlier in life. She also reported having manodepressive (bipolar) disorder. MRI of the brain, however, showed a single small frontal subcortical WML at age 58 years. She had a small infarct in the left superior cerebellar artery territory in the cerebellum with location and appearance consistent with embolic etiology, whereas no lacunar infarcts were observed. The brother of the index patient, age 55 years, was negative for the mutation. He had hypertension and reported high blood lipids and sleeping problems but had no history of headaches. MRI of the brain showed a small number of small, nonconfluent WMLs bifrontally in the subcortical and deep white matter, with a nonspecific, microvascular character. Another sister of the index patient, age 54 years, was negative for the mutation. She had migraine with aura and hypertension but had no MRI abnormalities. The half-brother (same mother) was not investigated for the mutation because the mutation was inherited on the index patient's father's side. A summary of the clinical picture of the family is provided in table e-1, in the online-only data supplement.

As some of the family members did not carry the *NOTCH3^A1604T^* mutation but still presented with WML, albeit of a somewhat different type, we decided to conduct WGS of 2 such family members, the mother and the older sister, and as control of the index patient, in search for other potential mutations associated with SVD. Although CADASIL is the most common monogenic form of SVD, mutations have also been identified in other genes, notably, in collagen type IV alpha1 (*COL4A1*), collagen type IV alpha2 (*COL4A2*), and Htra serine protease 1 (*HTRA1*) genes.^26^ *COL4A1* mutations cause familial vasculopathy, and the clinical features include ischemic stroke and intracerebral hemorrhage with lacunar infarction and microbleeds.^26,27^ Analysis of the WGS data for *COL4A1, COL4A2*, and *HTRA1* mutations revealed that the mother and older sister were heterozygous for a novel *COL4A1* missense mutation (c.4795 G>A; p.(Ala1599Thr)) (figure 1 and table e-1, links.lww.com/NXG/A415, in the online-only data supplement), whereas the index patient did not carry this mutation. None of the 3 family members carried mutations in the *HTRA1* gene, and the genome-wide association studies (GWAS) data confirmed that the index patient, but not the mother and older sister, was heterozygous for the *NOTCH3^A1604T^* mutation. Analysis by Sorting Tolerant from Intolerant (SIFT), PolyPhen, Align GVGD, and MutationTaster indicated that the *COL4A1* c.4795 G>A; p.(Ala1599Thr) mutation could be pathologic (data not shown). In conclusion, the genetic analysis reveals that the family harbors mutations in both the *NOTCH3* and *COL4A1* genes and that these mutations segregate with 2 different modes of WML with distinct clinical features.

### Reduced Activation of Notch Downstream Genes From the NOTCH3^A1604T^ Receptor

**Figure 3:**
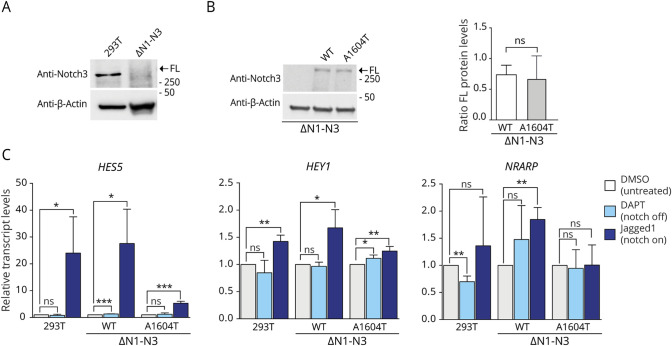
Reduced Activation of Notch Downstream Genes by the NOTCH3^A1604T^ Receptor (A) Western blot analysis of the inactivation of *NOTCH3* by CRISPR/Cas9 in the 293T(ΔN1-N3) cells (B). Western blot analysis showing the levels of full-length wild-type and A1604T NOTCH3 receptor in the 293T(ΔN1-N3)N3wt (wt) and 293T(ΔN1-N3)N3A1604T (A1604T) cell lines, respectively, compared with 293T(ΔN1-N3) cells as the control (left). β-Actin levels were used as loading controls. The positions of the FL form of the receptor are indicated. On the right: quantification of the full-length receptor in the 293T(ΔN1-N3)N3wt and 293T(ΔN1-N3)N3A1604T cell lines. (C). Analysis of messenger RNA (mRNA) expression from the *HES5, HEY1*, and *NRARP* genes in the 293T(ΔN1-N3)N3wt and 293T(ΔN1-N3)N3A1604T cell lines or in 293T cells as the control in response to activation of the wild-type or A1604T NOTCH3 receptor by Jagged1 (Notch on) or block of S3 cleavage by DAPT treatment (Notch off), as indicated. β-Actin levels were used as loading controls, and the positions of the FL form of the receptor are indicated. *GAPDH* mRNA expression was used for normalization. Values are significant at ***p < 0.001, **p < 0.01, and *p < 0.05. FL = full length.

To assess the functional consequences of the A1604T mutation with regard to Notch downstream signaling, we generated a 293T cell line in which the 3 expressed *NOTCH* genes (*NOTCH1*, *NOTCH2*, and *NOTCH3*) were inactivated by CRISPR/Cas9 genome editing (figure 3A; data for *NOTCH1* and *NOTCH2* ablation are shown in figure e-3, links.lww.com/NXG/A415). In the resulting 293T(ΔN1-N3) cell line, we stably introduced the full-length *NOTCH3* wild-type or *NOTCH3^A1604T^* receptor genes to generate 293T(ΔN1-N3)^N3wt^ and 293T(ΔN1-N3)^N3A1604T^ cell lines with similar expression levels (figure 3B).

We next analyzed how the NOTCH3^A1604T^ receptor affected the expression of Notch downstream genes (*HEY1*, *HES5*, and *NRARP*). Activation of Notch signaling was induced by culturing the 293T(ΔN1-N3)^N3wt^ and 293T(ΔN1-N3)^N3A1604T^ cell lines on the immobilized Jagged1 ligand,^28^ and Notch signaling was abrogated by treatment with the γ-secretase inhibitor DAPT. *HES5* expression was strongly upregulated in response to ligand activation in control 293T cells and NOTCH3 wild-type cells but more modestly in cells expressing *NOTCH3^A1604T^* (figure 3C). A similar pattern was observed for *HEY1*, although upregulation in response to ligand activation was less pronounced, and NRARP was upregulated only in the NOTCH3 wild-type cells but not in the cells expressing *NOTCH3^A1604T^* (figure 3C). In sum, these results suggest that NOTCH3^A1604T^ is a ligand-activated, signaling-competent, but hypomorphic form of the receptor.

### Aberrant Proteolytic Processing of the NOTCH3^A1604T^ Receptor

**Figure 4:**
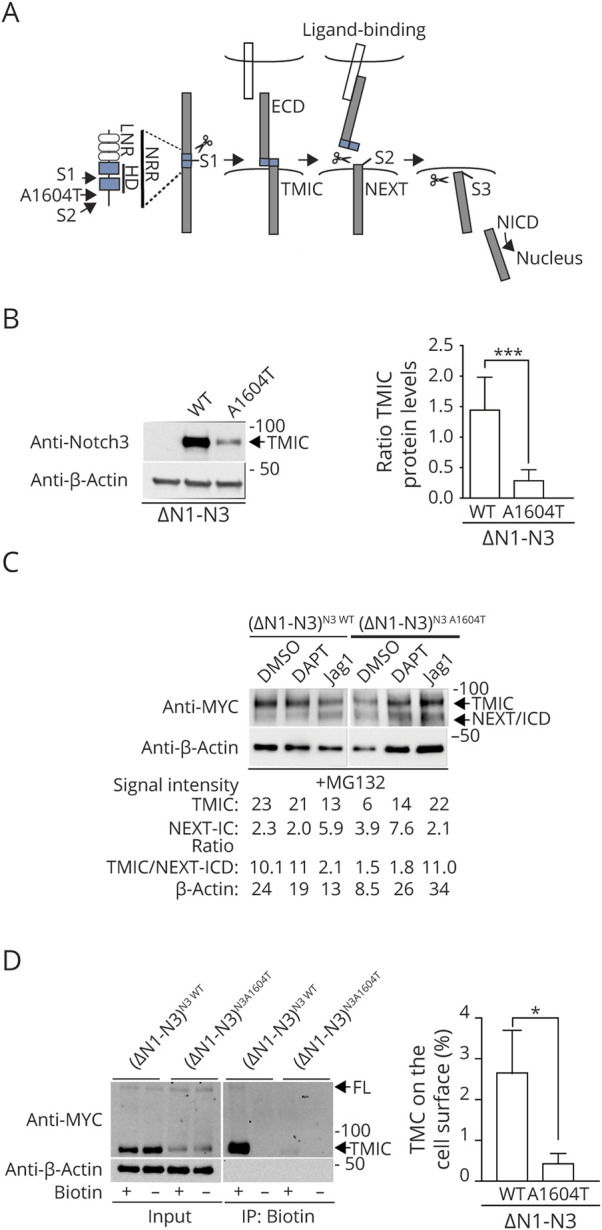
Aberrant Proteolytic Processing and Routing of the NOTCH3^A1604T^ Receptor (A) Schematic presentation of Notch receptor processing (S1, S2, and S3 cleavage and the ECD, TMIC, NEXT, and ICD moieties). The locations of the NRR the HD (in blue) and the Lin12/Notch repeats (LNR) are also depicted. (B). Western blot analysis of TMIC levels in the 293T(ΔN1-N3)N3wt (wt) and 293T(ΔN1-N3)N3A1604T (A1604T) cell lines (left) with quantification of the TMIC levels to the right. Note that the β-actin Western blot in figure 4B is the same as in figure 3B, i.e., the full-length NOTCH3 band in figure 3B and the TMIC band in figure 4B are derived from the same Western blot and thus share the same β-actin loading control. (C). Western blot analysis of TMIC and NEXT/ICD fragments from the 293T(ΔN1-N3)N3wt and 293T(ΔN1- N3)N3A1604T cell lines, following activation by ligand (Jagged1) or blockade of S3 cleavage (DAPT), as indicated. The signal intensity (as TMIC, NEXT-ICD, ratio TMIC/NEXT-ICD, and β-actin) for the Western blot is shown below. (D). Western blot analysis of biotin cell surface labeling of the NOTCH3 receptor, using an antibody to the NOTCH3 ICD (left). The protein amounts from input (input) and following immunoprecipitation for biotin (IP: Biotin) are shown for the 293T(ΔN1-N3)N3wt (wt) and 293T(ΔN1-N3)N3A1604T (A1604T) cell lines. To the right, the ratio of TMIC immunoprecipitated over TMIC input is shown. β-Actin levels were used as loading controls, and the positions of the full-length (FL), TMIC, and NEXT/ICD forms of the receptor are indicated. Values are significant at ***p < 0.001 and *p < 0.05. ECD = extracellular domain; HD = heterodimerization domain; ICD = intracellular domain; NRR = negative regulatory region; TMIC = transmembrane and intracellular.

To find whether proteolytic processing was altered in the NOTCH3^A1604T^ receptor, we assessed the amounts of processed intermediate forms of the wild-type and A1604T receptors. After site 1 (S1) cleavage by a furin-like convertase in the Golgi compartment, the Notch receptor appears at the cell surface as a bipartite, heterodimeric protein, with the ECD attached to the transmembrane portion of the receptor (TMIC) via noncovalent interactions between the 2 halves of the heterodimerization domain, which are generated by S1 cleavage (figure 4A). Ligand activation results in S2 cleavage by a disintegrin and metalloprotease (ADAM) proteases, which converts the transmembrane and intracellular domain (TMIC) into the Notch extracellular truncation (NEXT) moiety. NEXT in turn is a substrate for γ-secretase processing in the plasma membrane or endosomes (S3 cleavage), which releases the ICD (figure 4A).^14^ The TMIC (97 kDa) can be distinguished from NEXT/ICD (≈76 and ≈73 kDa, respectively) in Western blot experiments, whereas the NEXT is difficult to distinguish from the ICD because of their similar molecular weights.

We observed a strong reduction of the TMIC form of NOTCH3^A1604T^ in the Notch off state, that is, without ligand stimulation (figure 4B), despite similar levels of full-length receptors in the whole-cell lysate (figure 3B). We next assessed processing in the Notch on state by activating the NOTCH3 receptors via culturing the receptor-expressing cells on the immobilized Jagged1 ligand.^28^ To capture the ICD form, which is rapidly turned over in the cell, we performed the experiments in the presence of the proteasome inhibitor MG132, which blocks the degradation of ICD.^29^ For the NOTCH3 wild-type receptor, ligand activation by an immobilized Jagged1 ligand, as expected, led to a reduction in TMIC, accompanied by an accumulation of the NEXT/ICD band in the presence of MG132 (figure 4C). In contrast, for the NOTCH3^A1604T^ receptor, Jagged1 stimulation caused a noticeable increase in the TMIC over NEXT/ICD ratio (figure 4C). Stimulation by the immobilized Deltalike 4 (Dll4) ligand resulted in a weaker activation of Notch signaling and yielded a slightly weaker TMIC band and no discernible NEXT/ICD band (figure e-4, links.lww.com/NXG/A415). For comparison, we next analyzed a previously described *NOTCH3* receptor carrying a conventional cysteine-altering CADASIL mutation (R142C). Analysis of the NOTCH3^R142C^ receptor showed that the amount of TMIC was likewise reduced compared with a wild-type NOTCH3 receptor control (figure e-5), although the NOTCH3^R142C^ receptor retains a normal downstream signaling capacity.^30^

We next analyzed whether the aberrant processing of the receptor was reflected in the altered presence of the NOTCH3^A1604T^ receptor at the cell surface. In keeping with the results in figure 4B, the amount of TMIC was reduced in the cells expressing the NOTCH3^A1604T^ receptor, but the difference in biotinylated TMIC, after cell surface biotinylation and biotin immunoprecipitation, was even more profound, with large amounts of biotinylated, that is, cell surface-expressed, TMIC and a small amount of the full-length form from the cells expressing wild-type NOTCH3 compared with only minute quantities of TMIC from the NOTCH3^A1604T^ receptor-expressing cells (figure 4D).

**Figure 5:**
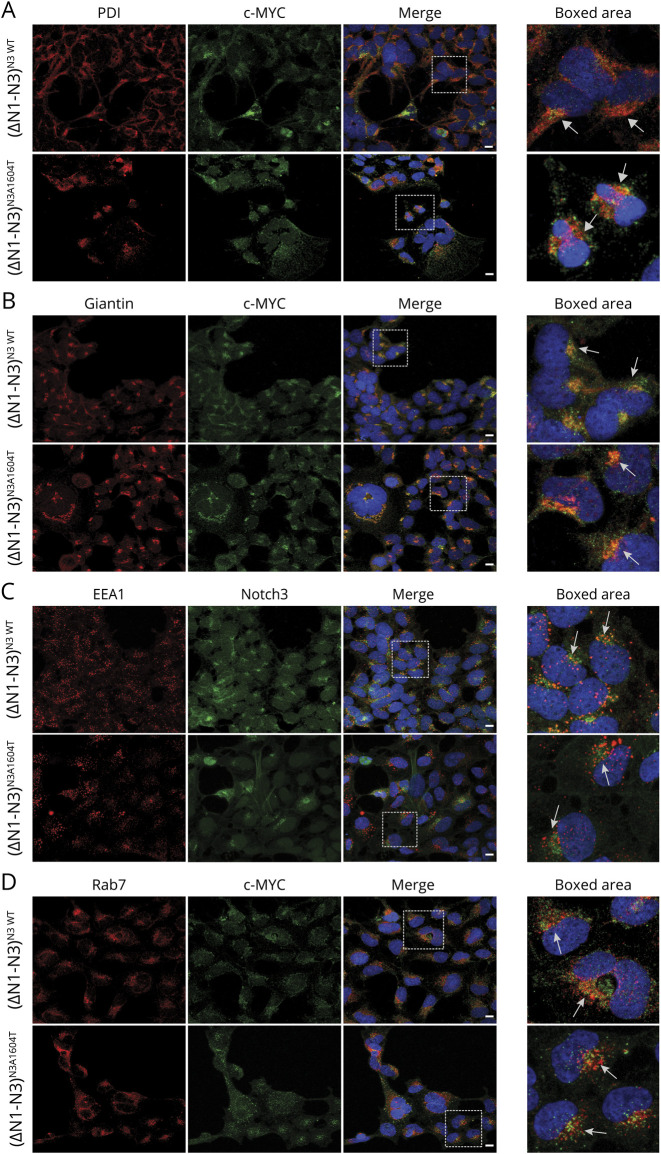
Intracellular Localization of the NOTCH3 Wildtype and A1604T Receptors (A-D) Immunocytochemistry for (A) endoplasmic reticulum (ER) (PDI; protein disulphide-isomerase), (B) Golgi (Giantin), (C,D) early and late endosomes (EEA1 and Rab7, respectively) are shown in red. Notch3 wild type or mutated receptor was stained with anti c-MYC or NOTCH3 antibody and shown in green. Scale bar: 10 mm.

**Figure 6:**
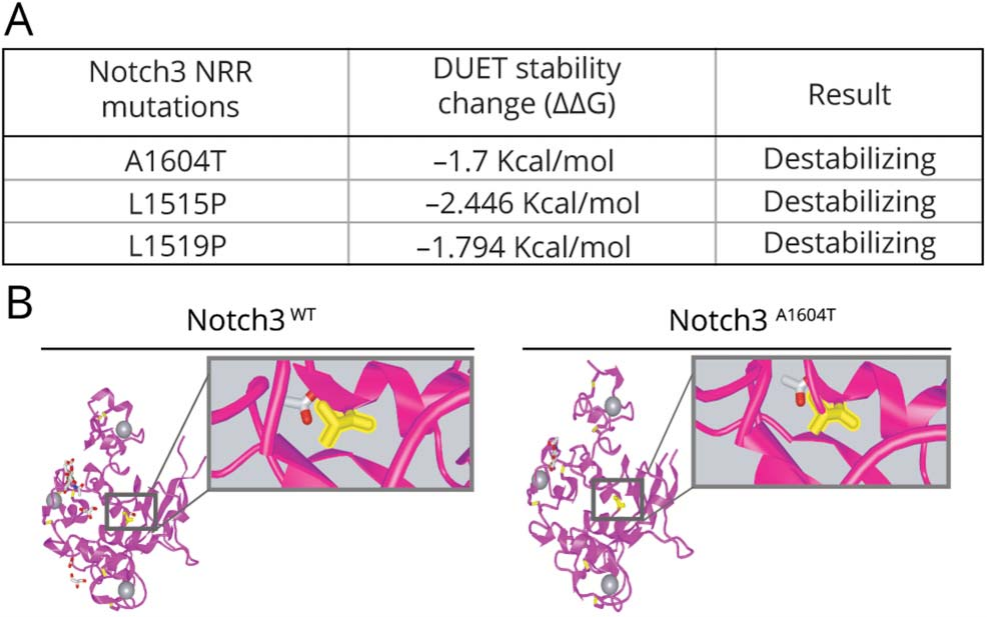
Predictions of Protein Stability and 3D Structure of NOTCH3 Mutations (A) Prediction of the protein stability change expressed in Kcal/mol for the A1604T, L1515P, and L1519P NOTCH3 mutations. (B) Predicted 3D structures of the NRR domain of the NOTCH3 wild-type and A1604T receptors. The alanine (left) and threonine (right) are visualized in the boxed area (see also figure e-7, links.lww.com/NXG/A415). NRR = negative regulatory region.

As the imbalance between the full-length and TMIC forms may indicate a problem in S1 or S2 processing, we next assessed the localization of the NOTCH3^A1604T^ receptor during intracellular routing. This was analyzed by costaining the wild-type or A1604T NOTCH3 receptors with markers for the endoplasmic reticulum (ER) (protein disulfide-isomerase [PDI]), Golgi (Giantin), and early and late endosomes (EEA1 and Rab7, respectively). Immunocytochemistry data from the 293T(ΔN1-N3)^N3wt^ and 293T(ΔN1-N3)^N3A1604T^ cell lines revealed no significant differences in distribution between the mutated and wild-type receptors and no overt aberrant accumulation in the ER or Golgi compartments, as judged by the extent of costaining with the PDI and Giantin markers (figure 5A and B). Similarly, we observed both wild-type and A1604T NOTCH3 receptors colocalizing with the EEA1 and Rab7 markers, indicating that both receptor forms are present in early and late endosomes (figure 5C and D). Because the A1604T mutation is localized to the HD region close to the S2 processing site (figure 4A), we next assessed the potential severity of the mutation from a conformation perspective. Using the DUET server,^25^ the A1604T mutation has a predicted stability change of −1.7 kcal/mol, a decrease similar to that observed for 2 other previously described missense mutations in the NOTCH3 HD region: the L1515P mutation linked to SVD (−2.4 kcal/mol) and the L1519P mutation linked to infantile myofibromatosis (−1.79 kcal/mol)^31-33^ (figure 6A). A depiction of the potential change in the 3D structure of NOTCH3 caused by the A1604T mutation is shown in figure 6B. Collectively, these data suggest that NOTCH3^A1604T^ receptor processing is altered and the data are compatible with the view that S2 rather than S1 processing is disturbed by the *NOTCH3^A1604T^* mutation.

### NOTCH3^A1604T^ Receptor-Expressing Cells Have Fewer Focal Adhesions

CADASIL-derived VSMC have larger size, slower proliferation rates, and an altered actin cytoskeleton compared with control VSMC.^34^ We observed a tendency toward slower growth of the NOTCH3^A1604T^ receptor-expressing cells compared with the control 293T(ΔN1-N3)^N3wt^ cells (figure e-6A, links.lww.com/NXG/A415). Cellular morphology was also different in cells expressing the NOTCH3^A1604T^ receptor compared with parental HEK293T cells and NOTCH3 wild-type–expressing cells (figure e-6B). Visualization of the actin cytoskeleton using phalloidin revealed an altered actin filament organization in the NOTCH3^A1604T^ receptor-expressing cells, with a more irregular filament distribution (figure e-6C). Furthermore, the 293T(ΔN1-N3)^N3A1604T^ cells exhibited an increased number of endpoints and a trend toward reduced number of actin filament branches (figure e-6C). There were also a reduced number of counts per cell for paxillin, which serves to adhere cells to the extracellular matrix, in the 293T(ΔN1-N3)^N3A1604T^ cells (figure e-6D). These data from 293T cells may suggest that the *NOTCH3^A1604T^* mutation causes a difference in cell adhesion properties, and it will be interesting to explore whether similar differences are observed also in VSMC.

## Discussion

In this report, we identified a family carrying a cysteine-sparing *NOTCH3^A1604T^* mutation. Two heterozygous carriers of the mutation presented with migraine and WML. WML distribution was distinct from what is usually observed in patients with CADASIL, with only mild or no involvement of the anterior temporal region and capsula externa. No lesions were observed in the basal ganglia. No microbleeds or infarcts and no overt atrophic changes were found.

In contrast to typical CADASIL, not all A1604T mutation carriers develop migraine and WML; a third sibling heterozygous for the mutation had headache but did not present with WML. Conversely, other family members, who were negative for the A1604T mutation, exhibited hypertension and neurologic problems including polyneuropathy and had WML, though of a more microangiopathic type. This may reflect a more severe form of the disease or a distinct type of disease, and in support of the latter possibility, we found that both the mother and older sister carried a novel missense mutation in the *COL4A1* gene (c.4795G>A; p.(Ala1599Thr)), which was considered pathologic. *COL4A1* mutations have been shown to cause SVD,^26,27^ and the microangiopathic changes observed in the mother and older sister are consistent with those observed in patients with *COL4A1* mutations. Together, these data show that 2 different mutations in genes known to be involved in SVD are found in this family and are associated with distinct clinical features. We cannot formally conclude that the *NOTCH3^A1604T^* mutation is causative for the observed migraine and WML in the 2 heterozygous siblings; it still remains a possibility that the mutation and symptoms are coincidental. The hypomorphic nature of the NOTCH3^A1604T^ receptor may, however, suggest that the vasculature is affected because loss of NOTCH3 function causes a gradual loss of VSMC in the mouse.^18,19^ Vascular impairment may in turn result in migraine and WML but with incomplete penetrance and possibly of a different type than in CADASIL, given the cysteine-sparing nature of the A1604T mutation. Other cysteine-sparing *NOTCH3* mutations have indeed been correlated with adverse effects. There are patients with CADASIL carrying a *NOTCH3^R75P^* mutation,^35,36^ and a patient with a *NOTCH3^L1515P^* mutation exhibited SVD but without GOM deposits or accumulation of the NOTCH3 protein.^31^ Of interest, the *NOTCH3^L1515P^* mutation caused aberrant receptor processing but, in contrast to the A1604T mutation, led to increased Notch signaling.^31^

The hypomorphic signaling behavior is an apparent contrast to the aberrant processing of the mutant receptor, which resulted in a reduced TMIC over NEXT/ICD ratio when proteasomal degradation is blocked by MG132. Of interest, a NEXT/ICD band appears also in the Notch off state, i.e., without ligand activation. This may be explained by either a problem in the S1 processing step or by the fact that the A1604T mutation causes enhanced and ligand-independent S2 processing. As problems in S1 processing likely occur in the Golgi compartment,^37^ one may surmise that S1 processing may lead to disturbed receptor routing through the ER and Golgi compartments and possibly also less subsequent routing through endosomes. The observation that the localization of the NOTCH3^A1604T^ receptor in the ER, Golgi, and endosome compartment is not significantly different from that of the wild-type NOTCH3 receptor may therefore argue against an S1 processing problem. The altered ratio between TMIC and NEXT/ICD between wild-type and NOTCH3^A1604T^, combined with the fact that the alanine-to-threonine transition at position 1604 in the HD domain may affect protein structure, also supports an S2 processing-related problem. Support for the hypothesis that mutations in the NOTCH3 HD domain can lead to aberrant processing and intracellular routing comes from analysis of 2 other mutations. Notably, a *NOTCH3^L1515P^* mutation produces a NOTCH3 receptor with enhanced signaling and secretion of the ECD and has been linked to cerebral SVD.^31^ Similarly, a *NOTCH3^L1519P^* mutation, which is found in a family with infantile myofibromatosis,^32^ generates a ligand-independent hyperactive form of NOTCH3.^33,38^ At this stage, however, we cannot formally exclude any of the hypotheses, and how the mutation may directly affect S2 processing remains to be tested. The conundrum of altered S2 processing, which appears to take place also in the absence of ligand activation, coupled with reduced signaling activity is likewise not yet understood, but the receptor may be incorrectly routed in the cell, possibly to some extent bypassing accumulation at the cell surface, and the reduced amounts of NOTCH3^A1604T^ receptor at the cell surface may indicate problems in intracellular routing.

In conclusion, our study reveals a family with migraine and WML carrying a cysteine-sparing hypomorphic *NOTCH3* mutation. This finding is relevant to the discussion on how dysregulated Notch signaling may more broadly play a role in cerebral SVDs and may inspire the search for novel *NOTCH* mutations in patients with neurologic and neurovascular pathologies, which are similar to but not completely typical of CADASIL.

## Glossary

ADAM: a disintegrin and metalloprotease
BSA: bovine serum albumin
CADASIL: cerebral autosomal dominant arteriopathy with subcortical infarcts and leukoencephalopathy
GOM: granular osmiophilic material
COL4A1: collagen type IV alpha1
COL4A2: collagen type IV alpha2
CRISPR/Cas9: clustered regularly interspaced short palindromic repeats/CRISPR-associated protein 9
DAPT: N-[N-(3,5-difluorophenacetyl)-L-alanyl]-S-phenylglycine t-butyl ester
ECD: extracellular domain
FL: full length
HTRA1: Htra serine protease 1
ICD: intracellular domain
NEXT: notch extracellular truncation
PBS: phosphate buffered saline
RT-qPCR: real-time quantitative PCR
S1: site 1
S2: site 2
S3: site 3
SCA: superior cerebellar artery
SVD: small vessel disease
TMIC: transmembrane and intracellular
VSMC: vascular smooth muscle cell
WGS: whole-genome sequencing
WML: white matter lesion
